# High genetic structure and low mitochondrial diversity in bottlenose dolphins of the Archipelago of Bocas del Toro, Panama: A population at risk?

**DOI:** 10.1371/journal.pone.0189370

**Published:** 2017-12-13

**Authors:** Dalia C. Barragán-Barrera, Laura J. May-Collado, Gabriela Tezanos-Pinto, Valentina Islas-Villanueva, Camilo A. Correa-Cárdenas, Susana Caballero

**Affiliations:** 1 Laboratorio de Ecología Molecular de Vertebrados Acuáticos LEMVA, Departamento de Ciencias Biológicas, Universidad de los Andes, Laboratorio J-202, Bogotá, Colombia; 2 Fundación Macuáticos Colombia, Medellín, Colombia; 3 Department of Biology, University of Vermont, Burlington, VT, United States of America; 4 Centro de Investigaciones del Mar y Limnología, Universidad de Costa Rica, San Jose, Costa Rica; 5 Coastal-Marine Research Group, INMS, Massey University, Auckland, New Zealand; 6 CONACYT, Universidad del Mar, Instituto de Genética, Ciudad Universitaria, Puerto Ángel, Distrito de San Pedro Pochutla, Oaxaca, México; 7 Facultad de Ingeniería y Ciencias Básicas, Departamento de Ciencias Naturales, Universidad Central, Bogotá, Colombia; 8 Departamento de Ciencias Básicas, Universidad de La Salle, Bogotá, Colombia; National Cheng Kung University, TAIWAN

## Abstract

The current conservation status of the bottlenose dolphin (*Tursiops truncatus*) under the IUCN is ‘least concern’. However, in the Caribbean, small and localized populations of the ‘inshore form’ may be at higher risk of extinction than the ‘worldwide distributed form’ due to a combination of factors including small population size, high site fidelity, genetic isolation, and range overlap with human activities. Here, we study the population genetic structure of bottlenose dolphins from the Archipelago of Bocas del Toro in Panama. This is a small population characterized by high site fidelity and is currently heavily-impacted by the local dolphin-watching industry. We collected skin tissue samples from 25 dolphins to study the genetic diversity and structure of this population. We amplified a portion of the mitochondrial Control Region (mtDNA-CR) and nine microsatellite loci. The mtDNA-CR analyses revealed that dolphins in Bocas del Toro belong to the ‘inshore form’, grouped with the Bahamas-Colombia-Cuba-Mexico population unit. They also possess a unique haplotype new for the Caribbean. The microsatellite data indicated that the Bocas del Toro dolphin population is highly structured, likely due to restricted movement patterns. Previous abundance estimates obtained with mark-recapture methods reported a small population of 80 dolphins (95% CI = 72–87), which is similar to the contemporary effective population size estimated in this study (*Ne* = 73 individuals; CI = 18.0 - ∞; 0.05). The combination of small population size, high degree of genetic isolation, and intense daily interactions with dolphin-watching boats puts the Bocas del Toro dolphin to at high risk of extinction. Despite national guidelines to regulate the dolphin-watching industry in Bocas del Toro and ongoing educational programs for tour operators, only in 2012 seven animals have died due to boat collisions. Our results suggest that the conservation status of bottlenose dolphins in Bocas del Toro should be elevated to ‘endangered’ at the national level, as a precautionary measure while population and viability estimates are conducted.

## Introduction

The bottlenose dolphin (*Tursiops truncatus*) is a cosmopolitan species [1] that despite its potential ability for long-distance movement, shows remarkable genetic differentiation at various geographic scales (e.g., [2, 3]). As with other delphinids, habitat specialization is considered an important driver for genetic differentiation [4, 5, 6, 7, 8]. Recent evidence suggests that in this species, habitat specialization may have occurred independently in different ocean basis, leading to different ecotypes [2, 9, 10]. For example, in the Western North Atlantic two forms or ecotypes, ‘offshore’ and ‘inshore’ show clear differences in their ecology, morphology, physiology, behavior, and genetic structure (e.g., [3, 4, 6, 9, 11, 12, 13, 14]). However, these differences are not evident in bottlenose dolphins from other regions [2, 15]. It appears that the ‘inshore form’ in the Western North Atlantic is the only ecotype clearly described so far that is genetically different to the rest (namely, the ‘offshore form’) [2, 9].

Some coastal populations of bottlenose dolphins live in small populations characterized by low genetic diversity and low gene flow between neighboring populations, suggesting local founder events at least in some areas or recent isolation (e.g., [3, 6, 8, 9, 13, 16, 17, 18, 19]). In some of these bottlenose dolphin populations’ males and females have differing home ranges, with females remaining within their natal groups, while males range further and visit different social units (e.g., [8, 20]). This fine scale genetic structuring in coastal bottlenose dolphins is evidenced in the remarkable variation of their social structure and feeding behaviors (e.g., [21, 22, 23, 24, 25]) across coastal populations. However, the high level of site fidelity, genetic isolation, and low abundance observed in some local populations [19, 26, 27], may impose significant challenges to these animals as they may not be able to respond to rapid changes in their habitat, particularly those caused by human activities (e.g. noise and chemical pollution, overfishing, entanglements, boat traffic) [28, 29, 30, 31]. In addition, several populations of bottlenose dolphins are affected by direct exploitation for exhibition purposes [e.g. 32], and others are regularly targeted by commercial dolphin-watching activities, which tend to have cumulative effects on their populations [26, 32, 33, 34].

Conservation priorities and ultimately, funding to monitor marine mammal populations is largely dependent on the IUCN conservation status of the species. Given their worldwide distribution, the bottlenose dolphin is classified by the IUCN as ‘least concern’ [35], with the exception of three populations: Fiordland in New Zealand (classified as ‘critically endangered’) [36], the Mediterranean Sea (classified as ‘vulnerable’) [37] and finally, the subspecies *T*. *truncatus* spp. *ponticus* from the Black Sea (classified as ‘endangered’) [38]. However, there are several distinct bottlenose dolphin coastal populations for which their conservation status is underestimated due to countries adopting the ‘least concern’ approach. This means that funding and research priorities are not directed to these populations. For example, the resident coastal bottlenose dolphin population of San Antonio Bay in Argentina is genetically isolated from neighboring populations [18] and presents and alarming population decline caused mainly by pollution and overfishing [19]. However, the Argentinian government still classifies this population as ‘least concern’ [19].

Like other coastal bottlenose dolphins, the dolphin population found in the Archipelago of Bocas del Toro in Panama is small. Preliminary estimates suggest a population of 80 dolphins (95% CI = 72–87) [34] and both sexes show high site fidelity [34, 39, 40, 41]. Analyses of mark-recapture using photo-identification, indicated this population consists of two interacting dolphin communities: one consisting of dolphins with high residency and a small home range located primarily within Dolphin Bay, and a second community consisting of highly mobile dolphins that show low residency [34]. While the transit of water boat taxis and other transport boats is common in the habitat of both communities, dolphins in the Dolphin Bay community are the once directly targeted by local dolphin-watching boats [34, 41, 42]. This dolphin community is preferred for dolphin-watching, due to their high predictability within the bay [34]. Since 2004, researchers have observed an exponential increase in the number of dolphin-watching boats visiting the bay [41]. In 2012, the highest number of boats interacting with dolphins was reported, with up to 39 boats interacting with the same group dolphin within a period of one hour [41]. That same year seven dolphins were reported dead due to boat collision [43]. Previous studies have showed that this increase in the number of boats is accompanied by an increase in engine noise levels [42] and has the potential to impact their acoustic space for communication [41], especially during foraging activities [41, 44], particularly in groups with nursing mothers [45].

Here, we evaluate the conservation status of the bottlenose dolphins of Bocas del Toro by estimating the genetic diversity and population structure using nine microsatellite markers and around 750 base pairs (bp) fragment of the mitochondrial DNA Control Region (mtDNA-CR). We test the hypothesis that there is a high degree of genetic distinctiveness in dolphins from Bocas del Toro in comparison to other bottlenose dolphin populations in the Caribbean, largely due to the small population size and high individual philopatry.

## Methods

**Fig 1:**
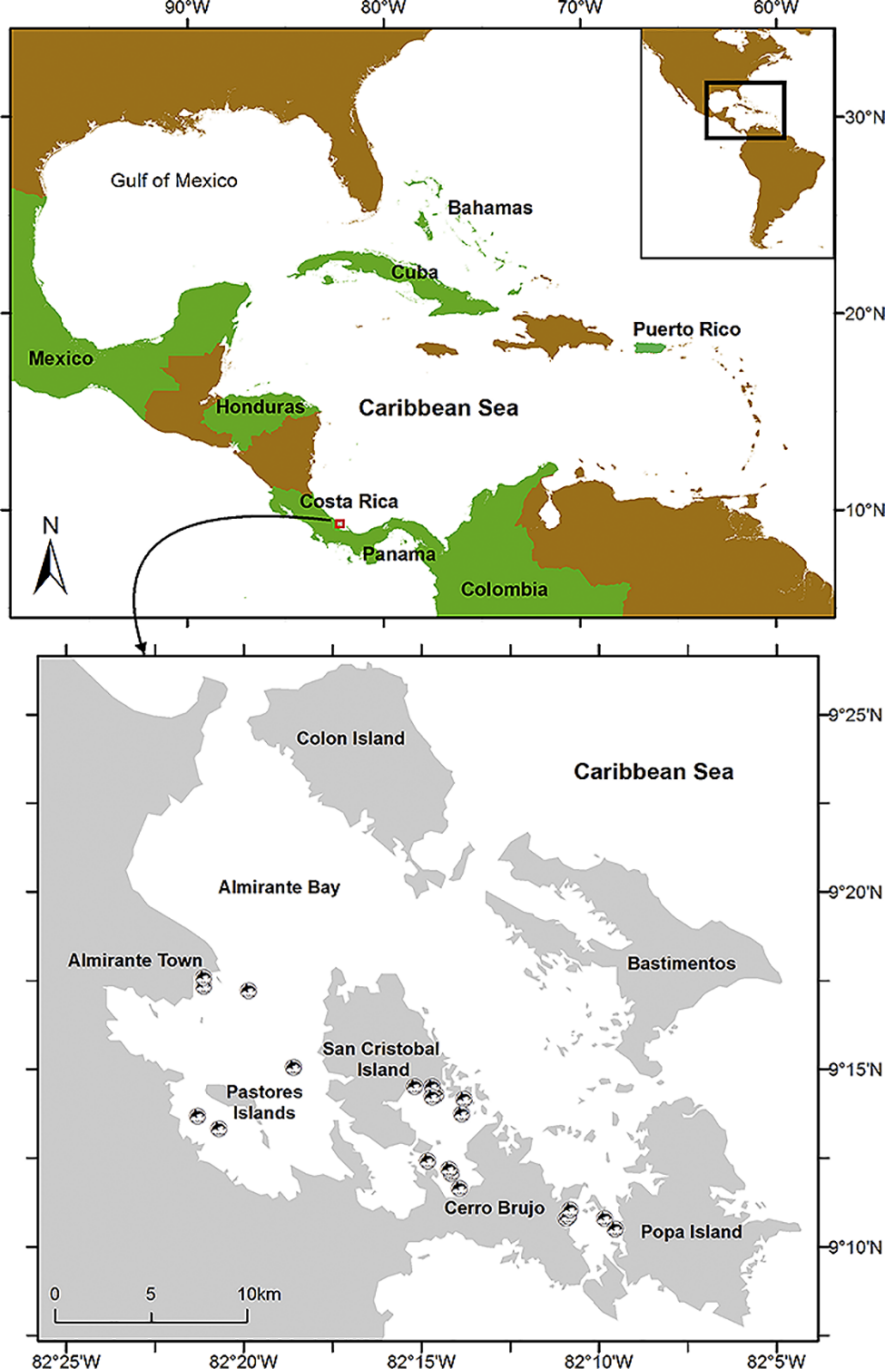
Location of Bocas del Toro (Panama) and the sampling sites including in this study (in green). These geographic locations also include The Bahamas, Colombia, Costa Rica, Cuba, Honduras, Mexico, and Puerto Rico. The map of Bocas del Toro below shows the position of common bottlenose dolphins sampled in the seven locations within the Archipelago, which include Almirante Bay, Dolphin Bay, Loma Partida, Pastores Islands, Popa, Shark Hole, and Tierra Oscura.

The Archipelago of Bocas del Toro (BDT) is located on the Caribbean coast of Panama (Fig 1). This area has several important marine ecosystems, including mangrove forests, sea grasses and coral reefs [46, 47, 48].

### Tissue samples collection

The methodology for sample collection was approved by the Smithsonian Tropical Research Institute IACUC (Institutional Animal Care and Use Committee; permit number 2011-1125-2014-06). Samples were collected with permission from the Autoridad Nacional del Ambiente–Panamá (ANAM; permits SC/A-11-12, SC/A-43-12, and SC/A-17-14).

Following Krützen et al. [49], tissue samples were obtained from wild dolphins by firing remote biopsy darts with a modified 0.22 veterinary rifle from a distance of approximately 10 m from the research boat [20, 49, 50, 51]. The biopsy darts have a hollow polycarbonate body and a small stainless steel biopsy tip (5 mm diameter, 9 mm length) [49, 52]. This system allows the collection of skin samples causing only a small wound, and short-term behavioral reactions [49 52]. Additionally, photographs of the dolphin biopsy sampled were collected to allow individual identification and linking demographic (e.g. site fidelity, residency) with genetic data (sex, lineage). This step was also included to avoid sampling the same individual repeatedly [18, 49]. A total of 24 biopsies were collected from seven locations within the Archipelago including Almirante Bay, Dolphin Bay, Loma Partida, Pastores Islands, Popa, Shark Hole, and Tierra Oscura (Fig 1). One additional sample was collected from a dead stranded dolphin in Bocastorito. We also included five samples from Gandoca-Manzanillo (Costa Rica), a bottlenose dolphin population located less than 35 km from Bocas del Toro. These five samples were collected by the Earthwatch Project ‘Dolphins of Costa Rica’ (authorization granted by Ministerio de Ambiente y Energía–Costa Rica, MINAE). All samples were conserved in 70% ethanol and stored at -20°C [53] for subsequent laboratory analysis.

### DNA Extraction, PCR analysis, sequencing, and molecular sexing

DNA was extracted from skin samples using the DNeasy kit (QIAGEN, Valencia, CA, USA). The portion of the mtDNA-CR (approximately 750 bp) was amplified by the polymerase chain reaction (PCR), using the primers pairs, t-Pro-whale M13Dlp1.5 (5′-TGTAAAACGACAGCCAGTTCACCCAAAGCTGRARTTCTA-3′) and Dlp8G (5′- GGAGTACTATG TCCTGTAACCA-3′). Amplification followed the protocol from Baker et al. [54]: an initial step of 94°C for 2 min, with 34 cycles of 2–4 repeat times of 30 s at 94°C, followed by 45 s at 55°C and an extension at 72°C, with a final extension after the last cycle of 10 min at 72°C. Each PCR mix of 30 μl reaction contained a 1X reaction buffer (10 mM Tris–HCl, 50 mM KCl, pH 8.3), 1.5 mM MgCl_2_, 200 μM dNTP, 2 units BSA, 0.5 μM of each primer, and 1 U Biolase DNA polymerase (Bioline USA). PCR products were purified following a Polietilenglicol protocol (PEG 20%), and DNA was sequenced using the Sanger sequencing method [55]. To examine the possibility of shared haplotypes among different locations (Bocas del Toro, Gandoca-Manzanillo, and Wider Caribbean), previously published sequences from Caballero and Islas-Villanueva et al. [3] were obtained from NCBI (Accession numbers: JN596281-JN596321). Finally, samples were sexed following the protocol proposed by Gilson et al. [56], amplifying the male-specific *SRY* gene and *ZFY/ZFX* genes of males and females as positive controls. The haplotypes sequences identified in this study were submitted to GenBank as accession number KX833116 for Bocas del Toro population, and KY817220 and KY817221 for individuals from Costa Rica. These two haplotypes from Costa Rica were successfully amplified in only two samples.

Analysis of eleven polymorphic microsatellite loci followed Caballero and Islas-Villanueva et al. [3]. Microsatellite loci included: *D08*, *D22* [57], *TexVet7*, *TexVet5* [58], *MK6*, *MK8*, *MK9* [59], *EV1* [60], *Tur48*, *Tur91* and *Tur117* [61], co-loaded for sizing in three lots for amplification. Each microsatellite locus was amplified separately in 10 μl mix PCR reactions. The thermocycling profile for each locus followed Caballero and Islas-Villanueva et al. [3]. Primers were fluorescently labeled for detection on an ABI 3500 automated sequencer using the internal ROX 500 size standard. All individuals were genotyped for at least nine loci. In order to allow for comparisons with previous microsatellite data from samples collected in the Caribbean [3], an internal control using previous data of 128 samples from six locations was used in all PCR amplifications and genotypification runs. In this data, *TexVet7* and *EV1* were not included; therefore these microsatellite loci were excluded in our analyses as well. Alleles were visualized and subsequently binned using GeneMapper Software (Life Technologies). These microsatellite data of the individual dolphins from Bocas del Toro and Costa Rica are available in Dryad (S1 Data).

### Data analyses

#### Mitochondrial DNA data analyses

To examine potential shared haplotypes, we used 40 mtDNA-CR sequences of bottlenose dolphins from the Wider Caribbean obtained from Caballero and Islas-Villanueva et al. [3] (available at NCBI accession numbers JN596281–JN596321). Frequency distribution of these haplotypes in the Caribbean locations are found in Table 1. All sequences were edited and aligned manually with sequences obtained from Bocas del Toro and Costa Rica, using the program GENEIOUS version 4.8.5 [62]. In order to understand the relationships among Bocas del Toro haplotypes with Costa Rican ones and previously described haplotypes from the Caribbean, a Neighbor-Joining tree was constructed using the software PAUP version 4.0 [63].

**Table 1:** *D-loop* haplotype frequency distribution among Caribbean bottlenose dolphin populations according to Caballero and Islas-Villanueva et al. [3]. New haplotypes reported in this study (*TruBOC* from Bocas del Toro, and *TtruCR1* and *TruCR2* from Costa Rica) are in bold.

Location	The Bahamas	Colombia	Cuba	Honduras	Jamaica	Mexico	Puerto Rico	Virgin Islands	Panama (BDT)	Costa Rica
	N = 15	N = 4	N = 65	N = 6	N = 1	N = 40	N = 26	N = 1	N = 25	N = 2
**Haplotypes**	***Inshore form***									
TtruCAR-A	6		36							
TtruCAR-L			1							
TtruCAR-D			1			9				
TtruCAR-E	9		1							
TtruCAR-JJ							1			
TtruCAR-F						3				
TtruCAR-AA						5				
TtruCAR-K			2			2				
TtruCAR-N			1							
TtruCARITA02						2				
TtruCAR-S			1							
TtruCAR-X						1				
TtruCAR-M			1							
TtruCAR-B			12			2	4	1		
TtruCAR-Z						1				
TtruCAR-DD						1				
TtruCAR-U						1				
TtruCAR-EE						1				
TtruCAR-FF						2				
TtruCAR-CC						1				
TtruCAR-V						2				
TtruCAR-BB						1				
TtruCAR-Q			1							
**Ttru-BOC**									25	
***Worldwide Distributed Form (WDF)***										
TtruCAR-C		3	4	4			5			
TtruCAR-MM		1								
TtruCAR-P			1							
TtruCAR-G				2						
TtruCAR-J			1							
TtruCAR-GG							1			
TtruCARQR1						2				
TtruCAR-H							9			
TtruCAR-II							1			
TtruCAR-KK							1			
TtruCAR-I							2			
TtruCAR-T					1					
TtruCAR-O			1							
TtruCAR-R			1							
TtruCAR-Y						3				
TtruCAR-W						1				
TtruCAR-HH							1			
TtruCAR-LL							1			
**Ttru-CR1**										1
**Ttru-CR2**										1

We assessed genetic subdivision among Caribbean populations with an analysis of molecular variance (AMOVA) [64] performed by ARLEQUIN version 3.5 [65], along with the pairwise comparison of population differentiation indices *F*_*ST*_ [66] and *φ*_*ST*_ between all the populations analyzed. The Tamura-Nei genetic distance model [67] was used to obtain *φ*_*ST*_ estimates. To conduct these analyses we only considered sampling regions with n ≥ 2. Therefore, eight regions were included (The Bahamas, Colombia, Costa Rica, Cuba, Honduras, Mexico, Panama-BDT, and Puerto Rico), and samples from Jamaica and the US Virgin Islands were excluded (n = 1) (Table 1).

#### Microsatellite data analysis

To determine the genetic structure of bottlenose dolphins from Bocas del Toro in the Caribbean, we compared our microsatellite results (*n* = 25) with nuclear data of samples from Costa Rica (*n* = 5) and previously published nuclear data from six different Caribbean geographic locations, including The Bahamas (*n* = 11), Colombia (*n* = 3), Cuba (*n* = 53), Honduras (*n* = 6), Mexico (*n* = 30), and Puerto Rico (*n* = 20) [3]. Overall, nine microsatellite loci were analyzed for a total of 153 individuals.

The program TANDEM was used to conduct binning of microsatellite data. This software is based on a heuristic search with the Nelder-Mead Downhill Simplex algorithm, and to calculate the number of alleles it applies a least-square minimization of rounding errors [68]. We used the software MICRO-CHECKER version 2.2.3 with Bonferroni correction for multiple comparisons in all loci, to evaluate the presence of null alleles, allele dropouts, and potential scoring errors due to stutter peaks [69].

To examine the genetic structure in Bocas del Toro and the Wider Caribbean, we used the software STRUCTURE version 2.3.4 [70], with a burn in period set to 10000 iterations. We determined the probability estimates using 100000 Markov chain Monte Carlo (MCMC) iterations. To infer the true *K* from the log probability of the data *LnP(D)* [71], we first conducted the runs with *K* set from 1 to 10 for each value of *K* with the admixture model and correlated frequencies in the software STRUCTURE. After, using the program STRUCTURE HARVESTER [72], we compare the log probability *LnP(D)* of different values for *K* using an ad hoc statistic *ΔK*, which calculates the second order rate of change of *Ln P(D)*. The corresponding values for each *K* were plotted to determine the uppermost level of population structure for our dataset.

We assessed genetic differentiation and diversity among population units defined previously by STRUCTURE using the software ARLEQUIN version 3.5 [65]. We calculated the observed heterozygosity (*H*_*o*_), the expected heterozygosity (*H*_*E*_), levels of polymorphism (100000 Markov chain, 100 dememorization steps), and the linkage disequilibrium (*LD*) (10000 permutations). We estimated the number of alleles (*N*_*A*_) and allelic richness (*AR*) per locus using the rarefaction test implemented in the software HP-RARE [73]. We tested deviation from HW equilibrium (*HWE*) using the Microsoft Excel add-in GENALEX software version 6.5 [74]. We assessed significance values using a sequential Bonferroni correction [75] as implemented in an EXCEL calculator version 1.1 [76]. We calculated pairwise *F*_*ST*_ and *R*_*ST*_ values for each population’s pair to estimate gene flow using the software GENALEX version 6.5. (1000 permutations) [74]. We also calculated global conventional *F*_*ST*_ and global corrected *F*_*ST*_ using the HIERFSTAT package in R version 3.3.3 [77]. To determine genetic subdivision among Caribbean populations, and to compare variations between and within groups, we performed an AMOVA [64] in the program ARLEQUIN version 3.5 [65].

To estimate the number of migrants per generation (*N*_*m*_) among pairs of population units of bottlenose dolphins in the Caribbean, we ran the software MIGRATE version 3.0.3 with microsatellite data. This software uses the coalescent approach of Markov chain Monte Carlo (MCMC) to run the maximum likelihood with all population units simultaneously [78, 79]. To evaluate solution convergence of the parameters obtained by MCMC, we ran multiple times ten short chains (500 used trees out of a sampled 10000) by three long chains (5000 used trees out of a sampled 100000), and lastly, a burn-in of 10000 [3].

Finally, we assessed the contemporary effective population size (*Ne*) for the Bocas del Toro population, using the bias-corrected version of the method of linkage disequilibrium [80, 81], as implemented in the program NeESTIMATOR version 2.01 (using polymorphism thresholds of 0.05 and 0.02) [82]. This software uses multi-locus diploid genotypes from population samples, and can estimate a reasonable contemporary *Ne* in small populations [82]. To conduct this analysis, only samples from adults were considered (two samples from male juveniles were omitted).

## Results

We collected 24 biopsy samples from bottlenose dolphins within the Archipelago of Bocas del Toro, including samples from the following locations: Almirante Bay (*n* = 3), Dolphin Bay (*n* = 10), Loma Partida (*n* = 3), Pastores Islands (*n* = 2), Popa (*n* = 2), Shark Hole (*n* = 2), and Tierra Oscura (*n* = 2) (Fig 1). Additionally, one sample was obtained from a dead stranded dolphin found in the mangrove in Bocastorito. Overall, there were 14 males (12 adults, two juveniles) and 11 females (all adults).

### MtDNA-CR results, population structure and ecotype classification

From the 25 samples collected in the Archipelago of Bocas del Toro, only one haplotype was identified (760 bp). We compared this haplotype to two sequences (760 bp and 540 bp) obtained from bottlenose dolphins of Gandoca-Manzanillo in Costa Rica, located only 35 km north of Bocas del Toro, and with 40 published bottlenose dolphins haplotypes found in multiple locations in the Wider Caribbean. Our results revealed that the haplotype found in Bocas del Toro is unique, as it is not found in any other bottlenose dolphin population in the Caribbean (Table 1). The differences are due to one transition, in position 192, (C→T).

**Fig 2:**
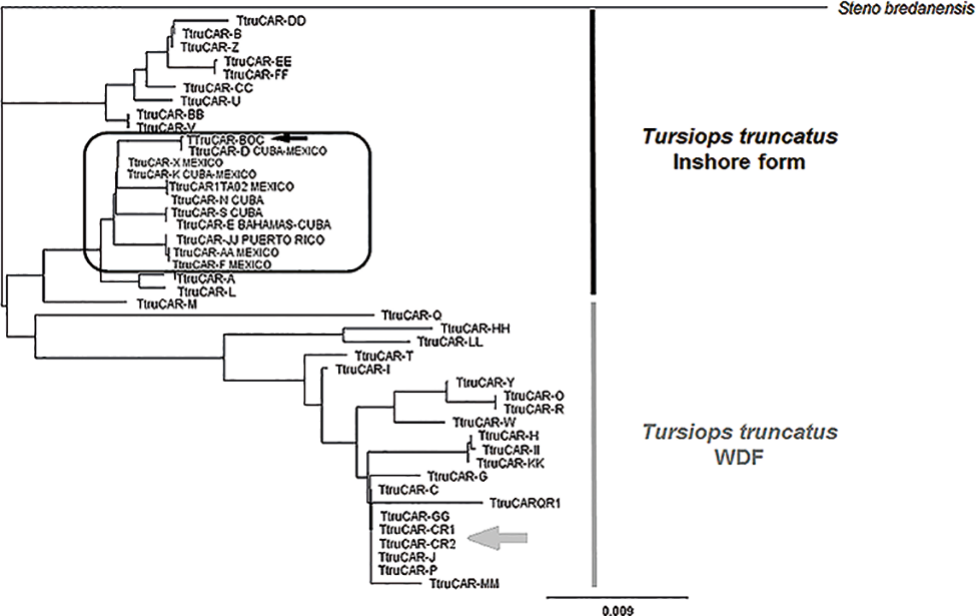
Neighbor-Joining reconstruction showing grouping of Wider Caribbean Control Region haplotypes. Black arrow indicates Bocas del Toro haplotype, which is grouped with haplotypes defined as belonging to the ‘inshore form’. These other haplotypes have been reported in The Bahamas (*TtruCAR-E*), Cuba (*TtruCAR-D*, *TtruCAR-E*, *TtruCAR-K*, *TtruCAR-N*, *TtruCAR-S*), Mexico (*TtruCAR-AA*, *TtruCAR-D*, *TtruCAR-F*, *TtruCAR-K*, *TtruCAR-X*, *TtruCAR1TA02*), and Puerto Rico (*TtruCAR-JJ*). Grey arrow indicates Costa Rican haplotypes, which is nested with WDF haplotypes.

The unique haplotype from Bocas del Toro was trimmed down to 320 bp to compare with previously published mtDNA-CR sequences. Comparisons were conducting Neighbor-Joining analyses that grouped the haplotype from Bocas del Toro in a clade with other haplotypes previously described as the ‘inshore form’ from The Bahamas (*TtruCAR-E*), Cuba (*TtruCAR-D*, *TtruCAR-E*, *TtruCAR-K*, *TtruCAR-N*, *TtruCAR-S*), Mexico (*TtruCAR-AA*, *TtruCAR-D*, *TtruCAR-F*, *TtruCAR-K*, *TtruCAR-X*, *TtruCAR1TA02*), and Puerto Rico (*TtruCAR-JJ*) (Fig 2; Table 1). Particularly, the haplotype *TtruCAR-D*, shared with Cuba a Mexico, has been suggested previously as ancestral [3]. The ‘inshore form’ was including 24 haplotypes (see Table 1) from The Bahamas, Cuba, Mexico, Puerto Rico, Panama-BDT, and Virgin Islands. Most of these dolphin populations shared haplotypes (Table 1), suggesting some degree of past or present connectivity among them.

The haplotype from Bocas del Toro was not shared with any other population. In contrast, the two haplotypes from Gandoca-Manzanillo (Costa Rica) were grouped with ‘worldwide distributed form’ (WDF) haplotypes from the Caribbean (see Fig 2; Table 1). Specifically, they nested with WDF haplotypes from Colombia (*TtruCAR-MM*), Cuba (*TtruCAR-J*, *TtruCAR-P*), Honduras (*TtruCAR-G*), Mexico (*TtruCAR-QR1*), and Puerto Rico (*TtruCAR-GG*). In addition, the Costa Rican haplotypes nested with the haplotype *TtCAR-C*, an ancestral WDF haplotype related to WDF haplotypes from Madeira [3], which is also shared with bottlenose dolphins from Colombia, Cuba, Honduras, and Puerto Rico (Fig 2; Table 1).

In the analysis of population structure, we defined two population units, where Bocas del Toro (BDT) was grouped with other subpopulations: BDT-Bahamas-Colombia-Cuba-Mexico, meanwhile Costa Rica was grouped with Honduras and Puerto Rico (Table 2). Significant population differentiation (*P* = 0.000) was found among both population units at the *F*_*ST*_ and *Φ*_*ST*_ levels (Table 2). In general, these populations units showed a differentiation less significant (*P < 0*.*1*) since we found a value of *Ф*_*CT*_ = 0.205 (*P* = 0.052), *Ф*_*ST*_ = 0.427 (*P* = 0.000), and the *Ф*_*SC*_ = 0.279 (*P* = 0.000).

**Table 2:** Population differentiation of *Tursiops truncatus* between pairwise populations in the Caribbean obtained with mtDNA-CR. High and significant values are indicated in bold and the *P-value* is shown below each value (*P-values* were obtained after 1000 permutations). *Ф*_*ST*_ values are indicated below the diagonal. *F*_*ST*_ values are above diagonal.

*F*_*ST*_	BDT-Bahamas-Colombia-Cuba-Mexico	CostaRica-Honduras-PuertoRico
*Ф*_*ST*_	N = 149	N = 34
BDT-Bahamas-Colombia-Cuba-Mexico	-	**0.133**
		(0.000)
CostaRica-Honduras-PuertoRico	**0.299**	-
	(0.000)	

### Microsatellite results

#### Population structure

**Fig 3:**
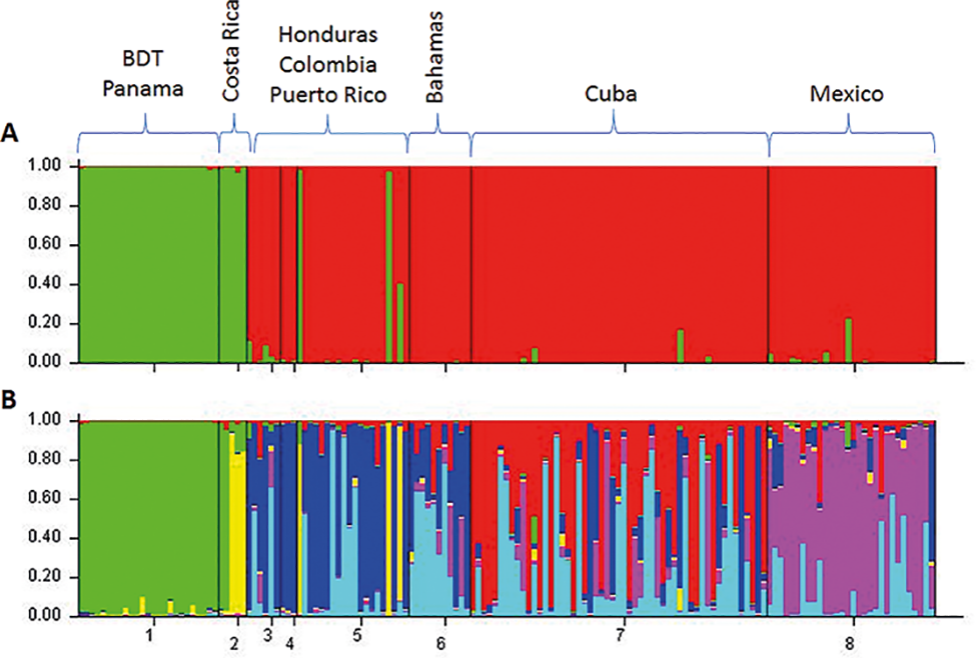
Barplot of the likelihood (Y-axis) of each individual’s (X-axis) assignment to a particular population units according to *ΔK* method [64] (*K* = 2, figure a). Based on four population units identified in the Caribbean in a previous work [3], and in the assumption that Bocas del Toro (Panama) and Gandoca-Manzanillo (Costa Rica) belong to two distinct population units, we also include *K = 6* (figure b). In both figures Bocas del Toro consists of one discrete population unit.

Based on the assumption that Caribbean bottlenose dolphins have a common ancestor, we performed the STRUCTURE analysis under the admixture model with correlated frequencies, in order to assess the number of units (*K*) present in the Caribbean. The evaluation of the *K* values using the *ΔK* method produced a clear peak at *K* = 2 (S1 Fig, S1 Table) in which Bocas del Toro was identified as one population unit (Fig 3A), and therefore, the software identified *K* = 2 as the most likely number of groups present in the data (*ΔK* = 267.317) (Fig 3A). However, a previous work by Caballero and Islas-Villanueva et al. [3] reported to *K* = 4 as the number of populations units present in the Caribbean according to the same microsatellite data (without including Bocas del Toro and Costa Rican samples). Based on four population units identified in this previous work (Bahamas; Colombia-Honduras-PuertoRico; Cuba; and Mexico), and in order to identify population structure between the Bocas del Toro population and individuals from Costa Rica, we assessed the population structure analyses in *K* = 6, in which Bocas del Toro (Panama) and Gandoca-Manzanillo (Costa Rica) belong to two distinct population units (Fig 3B). In Fig 3B (*K* = 6), where the *X* axis corresponds to each individual, it is notable that the Bocas del Toro cluster conforms a discrete population unit. This plot also shows that the Bocas del Toro population unit is highly differentiated from other population units and that individuals do not appear to maintain present gene flow with other dolphin population in the Caribbean, including Gandoca-Manzanillo, and if they do, it is minimal.

The results from the MIGRATE analysis indicated that there was only one migrant per generation from Bocas del Toro to the neighbor population in Gandoca-Manzanillo (*N*_*m*_ = 1.070) (Table 3), and rates of reverse migration are almost nil (*N*_*m*_ = 0.132), suggesting asymmetrical migration. Individuals from Gandoca-Manzanillo also showed low rates of migration toward other locations in the Caribbean. While both Bocas del Toro and Gandoca-Manzanillo dolphins show low migration rates, other dolphin populations in the Caribbean seem to maintain connectivity with at least one migrant per generation (Table 3). For instance, the Colombia-Honduras-PuertoRico population unit receives migrants from The Bahamas and Cuba (*N*_*m*_ = 1.113 and *N*_*m*_ = 1.133, respectively). Similarly, Colombia-Honduras-PuertoRico, The Bahamas, and Mexico population units contribute one migrant per generation towards Cuba (*N*_*m*_ = 1.526, *N*_*m*_ = 1.605, and *N*_*m*_ = 1.894, respectively), and the Mexico population unit receives migrants from Cuba (*N*_*m*_ = 1.598). Hence, all populations units in the Caribbean show a certain level of connectivity, except for Bocas del Toro and Gandoca-Manzanillo.

**Table 3:** Number of migrants per generation (*N*_*m*_) estimated for Caribbean *Tursiops truncatus* population units (credibility interval at 95%). *N*_*m*_ >1 are in bold.

Receiving population n	Panama (BDT)	Costa Rica	Colombia-Honduras-PuertoRico	The Bahamas	Cuba	Mexico
Source population						
Panama (BDT)	-	**1.070**	0.216	0.161	0.145	0.153
		(0.040–0.381)	(0.026–0.092)	(0.100–0.107)	(0.100–0.107)	(0.018–0.054)
Costa Rica	0.132	-	0.212	0.195	0.158	0.157
	(0.002–0.010)		(0.026–0.090)	(0.122–0.131)	(0.109–0.117)	(0.019–0.055)
Colombia-Honduras-PuertoRico	0.031	0.143	-	0.733	**1.526**	0.861
	(0.0005–0.509)	(0.005–0.051)		(0.456–0.490)	(1.054–1.129)	(0.103–0.303)
The Bahamas	0.029	0.146	**1.113**	-	**1.605**	0.670
	(0.0005–0.470)	(0.006–0.052)	(0.136–0.471)		(1.110–1.188)	(0.080–0.236)
Cuba	0.029	0.143	**1.133**	0.797	-	**1.598**
	(0.0005–0.480)	(0.005–0.051)	(0.138–0.480)	(0.496–0.532)		(0.192–0.562)
Mexico	0.028	0.149	0.736	0.747	**1.894**	-
	(0.0004–0.470)	(0.006–0.053)	(0.090–0.311)	(0.465–0.499)	(1.309–1.401)	

In general, global conventional *F*_*ST*_ (0.219, *P* = 0.000) and corrected *F*_*ST*_ (0.232, *P* = 0.000) values showed a population structure among bottlenose dolphins in the Wider Caribbean. The calculated pairwise population differentiation indices *F*_*ST*_ and *R*_*ST*_ for all six proposed dolphin populations are described in Table 4, and show that for the most part *R*_*ST*_ values are higher than *F*_*ST*_ values. This suggests an older differentiation among dolphin populations with some recent genetic connectivity [3]. Particularly for Bocas del Toro, *F*_*ST*_ and *R*_*ST*_ indicate that the Bocas del Toro population is differentiated from the other Caribbean dolphin populations, suggesting a certain degree of isolation. The other dolphin populations in the Caribbean show relatively low but significant *F*_*ST*_ and *R*_*ST*_ values. However, all these populations units did not show strong differences in *F*_*ST*_ and *R*_*ST*_ values, probably because some populations still present some migration (see Table 3).

**Table 4:** Population differentiation of *Tursiops truncatus* between pairwise populations in the Caribbean obtained with nine microsatellites. High and significant values are indicated in bold and the *P-value* is shown below each value (*P-values* were obtained after 10000 permutations). *R*_*ST*_ values are indicated below the diagonal. *F*_*ST*_ values are above diagonal. Degrees of significance: * 0.01 and ** 0.001.

*F*_*ST*_	Panama (BDT)	Costa Rica	The Bahamas	Colombia-Honduras-PuertoRico	Cuba	Mexico
*R*_*ST*_						
Panama (BDT)	-	0.149** (0.001)	0.411** (0.000)	0.366** (0.000)	0.382** (0.001)	0.373** (0.001)
Costa Rica	0.138* (0.006)	-	0.361** (0.001)	0.324** (0.001)	0.339** (0.001)	0.296** (0.001)
The Bahamas	0.464** (0.001)	0.712** (0.001)	-	0.048** (0.002)	0.065** (0.001)	0.134** (0.001)
Colombia-Honduras-PuertoRico	0.477** (0.001)	0.711** (0.001)	0.120** (0.052)	-	0.071** (0.001)	0.148** (0.001)
Cuba	0.374**(0.001)	0.587** (0.001)	0.048* (0.021)	0.080** (0.001)	-	0.107** (0.001)
Mexico	0.482** (0.001)	0.687**(0.001)	0.225** (0.001)	0.223** (0.001)	0.109**(0.001)	-

#### *Ne* estimation

The contemporary effective population size (*Ne*) was estimated for the Bocas del Toro population using minimum allele frequencies of 0.05 and 0.02. The *Ne* varied from a low of 73 (CI = 18.0 - ∞; 0.05) to a high of 122 (CI = 22.3 - ∞; 0.02). Despite *Ne* showing a wide confidence interval (CI), it is a good indicator of number of adults reproductively contributing to the Bocas del Toro dolphin population [81, 83].

#### Genetic diversity

Levels of genetic variation were measured by allelic richness (*AR*), number of alleles (*N*_*A*_), and expected heterozygosity (*H*_*E*_). We obtained genetic diversity values for nine microsatellites loci from the six populations units analyzed along with deviations from *HWE* (Table 5). After Bonferroni correction (*P-value* = 0.001562), dolphin populations in Colombia-Honduras-PuertoRico and Cuba showed three loci out of *HWE*, meanwhile Bocas del Toro showed one locus (Table 5). Samples from Cuba and Puerto Rico, which showed more loci out of *HWE*, came from aquariums and stranded animals, respectively [3]. Because the origin of these dolphins is not clear, it is possible that these samples came from more than one breeding unit (possibly transient WDF males mating with ‘inshore form’ females [3]), decreasing heterozygosity (Wahlund effect) [84], therefore generating a confounding effect in these results [3]. In contrast, dolphin populations in The Bahamas, Mexico, and individuals from Costa Rica did not show loci out of *HWE* (Table 5).

**Table 5:** Genetic diversity values for nine microsatellites loci in six *Tursiops truncatus* populations units analyzed (Panama, Costa Rica, The Bahamas, Colombia-Honduras-PuertoRico, Cuba, and Mexico). For each location, we include sample size (*n*). For each locus we include: total number of alleles (*N*_*A*_), allelic richness (*AR*), observed (*H*_*O*_), and expected (*H*_*E*_) heterozygosity. Loci out of *HWE* after Bonferroni correction (0.001562) are in bold.

	Panama		Costa Rica		The Bahamas		Colombia-Honduras-		Cuba		Mexico	
Locus	(Bocas del Toro—BDT)		(Gandoca-Manzanillo)		*n* = 11		Puerto Rico		*n* = 53		*n* = 30	
	*n* = 25		*n* = 5				*n* = 29					
*D08*	*N*_*A*_ = 4	*AR =* 1.531	*N*_*A*_ = 2	1.833	*N*_*A*_ = 2	*AR =* 1.809	*N*_*A*_ = 5	*AR =* 2.174	***N***_***A***_ **= 5**	***AR =* 1.529**	*N*_*A*_ = 7	*AR =* 2.473
*N*_*A*_ = 8	*H*_*O*_ = 0.160	*H*_*E*_ = 0.258	*H*_*O*_ = 0.600	*H*_*E*_ = 0.467	*H*_*O*_ = 0.636	*H*_*E*_ = 0.454	*H*_*O*_ = 0.444	*H*_*E*_ = 0.544	***H***_***O***_ **= 0.170**	***H***_***E***_ **= 0.258**	*H*_*O*_ = 0.600	*H*_*E*_ = 0.654
	*P* = 0.084		*P* = 0.338		*P* = 0.480		*P* = 0.016		***P* = 0.000**		*P* = 0.994	
*D22*	*N*_*A*_ = 5	*AR =* 2.590	*N*_*A*_ = 2	*AR =* 2.000	*N*_*A*_ = 5	*AR =* 2.398	*N*_*A*_ = 8	*AR =* 2.139	*N*_*A*_ = 9	*AR =* 2.497	*N*_*A*_ = 8	*AR =* 3.052
*N*_*A*_ = 12	*H*_*O*_ = 0.792	*H*_*E*_ = 0.701	*H*_*O*_ = 0.000	*H*_*E*_ = 0.667	*H*_*O*_ = 0.818	*H*_*E*_ = 0.623	*H*_*O*_ = 0.414	*H*_*E*_ = 0.522	*H*_*O*_ = 0.620	*H*_*E*_ = 0.671	*H*_*O*_ = 0.700	*H*_*E*_ = 0.818
	*P* = 0.706		*P* = 0.334		*P* = 0.883		*P* = 0.010		*P* = 0.582		*P* = 0.134	
*TexVet5*	*N*_*A*_ = 5	*AR =* 2.642	*N*_*A*_ = 3	*AR =* 3.000	*N*_*A*_ = 3	*AR =* 2.420	*N*_*A*_ = 4	*AR =* 2.590	*N*_*A*_ = 5	*AR =* 2.695	*N*_*A*_ = 5	*AR =* 2.308
*N*_*A*_ = 8	*H*_*O*_ = 0.880	*H*_*E*_ = 0.721	*H*_*O*_ = 0.500	*H*_*E*_ = 0.833	*H*_*O*_ = 0.818	*H*_*E*_ = 0.671	*H*_*O*_ = 0.517	*H*_*E*_ = 0.706	*H*_*O*_ = 0.680	*H*_*E*_ = 0.727	*H*_*O*_ = 0.433	*H*_*E*_ = 0.595
	*P* = 0.514		*P* = 0.334		*P* = 0.757		*P* = 0.077		*P* = 0.291		*P* = 0.060	
*MK6*	*N*_*A*_ = 4	*AR =* 1.819	*N*_*A*_ = 3	*AR =* 2.414	*N*_*A*_ = 7	*AR =* 2.828	***N***_***A***_ **= 6**	***AR =* 2.699**	*N*_*A*_ = 6	*AR =* 3.115	*N*_*A*_ = 6	*AR =* 2.883
*N*_*A*_ = 11	*H*_*O*_ = 0.250	*H*_*E*_ = 0.409	*H*_*O*_ = 0.250	*H*_*E*_ = 0.679	*H*_*O*_ = 0.818	*H*_*E*_ = 0.753	***H***_***O***_ **= 0.360**	***H***_***E***_ **= 0.717**	*H*_*O*_ = 0.788	*H*_*E*_ = 0.839	*H*_*O*_ = 0.733	*H*_*E*_ = 0.781
	*P* = 0.108		*P* = 0.086		*P* = 0.990		***P* = 0.000**		*P* = 0.429		*P* = 0.348	
*MK8*	***N***_***A***_ **= 4**	***AR =* 2.670**	*N*_*A*_ = 3	*AR =* 2.000	*N*_*A*_ = 5	*AR =* 2.994	*N*_*A*_ = 7	*AR =* 2.704	***N***_***A***_ **= 7**	***AR =* 2.893**	*N*_*A*_ = 6	*AR =* 2.829
*N*_*A*_ = 11	***H***_***O***_ **= 0.800**	***H***_***E***_ **= 0.731**	*H*_*O*_ = 0.250	*H*_*E*_ = 0.464	*H*_*O*_ = 0.556	*H*_*E*_ = 0.810	*H*_*O*_ = 0.607	*H*_*E*_ = 0.729	***H***_***O***_ **= 0.711**	***H***_***E***_ **= 0.778**	*H*_*O*_ = 0.444	*H*_*E*_ = 0.758
	***P* = 0.000**		*P* = 0.143		*P* = 0.078		*P* = 0.010		***P* = 0.000**		*P* = 0.032	
*MK9*	*N*_*A*_ = 4	*AR =* 2.205	*N*_*A*_ = 3	*AR =* 2.800	*N*_*A*_ = 4	*AR =* 2.546	***N***_***A***_ **= 6**	***AR =* 2.680**	***N***_***A***_ **= 7**	***AR =* 2.624**	*N*_*A*_ = 7	*AR =* 2.657
*N*_*A*_ = 9	*H*_*O*_ = 0.625	*H*_*E*_ = 0.579	*H*_*O*_ = 0.667	*H*_*E*_ = 0.800	*H*_*O*_ = 0.778	*H*_*E*_ = 0.699	***H***_***O***_ **= 0.259**	***H***_***E***_ **= 0.726**	***H***_***O***_ **= 0.490**	***H***_***E***_ **= 0.686**	*H*_*O*_ = 0.655	*H*_*E*_ = 0.712
	*P* = 0.062		*P* = 0.463		*P* = 0.739		***P* = 0.000**		***P* = 0.000**		*P* = 0.319	
*Tur117*	*N*_*A*_ = 3	*AR =* 2.376	*N*_*A*_ = 3	*AR =* 2.643	*N*_*A*_ = 2	*AR =* 1.222	*N*_*A*_ = 5	*AR =* 1.472	*N*_*A*_ = 5	*AR =* 2.045	*N*_*A*_ = 5	*AR =* 2.360
*N*_*A*_ = 6	*H*_*O*_ = 0.560	*H*_*E*_ = 0.654	*H*_*O*_ = 0.250	*H*_*E*_ = 0.536	*H*_*O*_ = 0.111	*H*_*E*_ = 0.111	*H*_*O*_ = 0.103	*H*_*E*_ = 0.197	*H*_*O*_ = 0.510	*H*_*E*_ = 0.496	*H*_*O*_ = 0.483	*H*_*E*_ = 0.628
	*P* = 0.327		*P* = 0.424		*P* = 1.000		*P* = 0.556		*P* = 0.384		*P* = 0.108	
*Tur91*	*N*_*A*_ = 4	*AR =* 2.132	*N*_*A*_ = 4	*AR =* 2.857	*N*_*A*_ = 2	*AR =* 1.554	***N***_***A***_ **= 4**	***AR =* 2.380**	*N*_*A*_ = 4	*AR =* 2.361	*N*_*A*_ = 5	*AR =* 2.237
*N*_*A*_ = 10	*H*_*O*_ = 0.522	*H*_*E*_ = 0.554	*H*_*O*_ = 0.750	*H*_*E*_ = 0.786	*H*_*O*_ = 0.333	*H*_*E*_ = 0.294	***H***_***O***_ **= 0.125**	***H***_***E***_ **= 0.627**	*H*_*O*_ = 0.458	*H*_*E*_ = 0.624	*H*_*O*_ = 0.536	*H*_*E*_ = 0.584
	*P* = 0.133		*P* = 1.000		*P* = 0.549		***P* = 0.000**		*P* = 0.090		*P* = 0.490	
*Tur48*	*N*_*A*_ = 2	*AR =* 1.226	*N*_*A*_ = 2	*AR =* 1.993	*N*_*A*_ = 4	*AR =* 2.176	*N*_*A*_ = 4	*AR =* 2.123	*N*_*A*_ = 4	*AR =* 1.561	*N*_*A*_ = 2	*AR =* 1.330
*N*_*A*_ = 5	*H*_*O*_ = 0.120	*H*_*E*_ = 0.115	*H*_*O*_ = 0.000	*H*_*E*_ = 0.533	*H*_*O*_ = 0.556	*H*_*E*_ = 0.542	*H*_*O*_ = 0.560	*H*_*E*_ = 0.528	*H*_*O*_ = 0.280	*H*_*E*_ = 0.282	*H*_*O*_ = 0.111	*H*_*E*_ = 0.171
	*P* = 0.950		*P* = 0.199		*P* = 0.272		*P* = 0.167		*P* = 0.993		*P* = 0.183	
Mean *H*_*O*_ and *H*_*E*_	*H*_*O*_ = 0.52316		*H*_*O*_ = 0.41852		*H*_*O*_ = 0.60269		*H*_*O*_ = 0.38053		*H*_*O*_ = 0.52535		*H*_*O*_ = 0.52588	
heterozigosity	*H*_*E*_ = 0.52463		*H*_*E*_ = 0.66429		*H*_*E*_ = 0.55108		*H*_*E*_ = 0.59173		*H*_*E*_ = 0.59612		*H*_*E*_ = 0.63385	

Microsatellite expected heterozygosity (*H*_*E*_) values were similar among the six populations, with Bocas del Toro and The Bahamas showing slightly higher values. Particularly for the Bocas del Toro dolphin population, heterozygosity values were moderate and similar for most of the loci (Table 5), and *H*_*E*_ was higher than *H*_*o*_ for four loci (*D08*, *MK6*, *Tur91*, and *Tur117*; Table 5). These differences could suggest slight inbreeding in the most recent generation of mating (positive *F*_*IS*_) or selection against heterozygotes [84]; however, we calculated the coefficient of local inbreeding (*F*_*IS*_) for the Bocas del Toro population, and although it was positive, it was not significant (*F*_*IS*_ = 0.154; *P >* 0.05). No significant differences between *H*_*E*_ and *H*_*o*_ were found (*P* > 0.05), meaning that there appear to be no significant reduction of diversity. Nevertheless, it is possible that we cannot detect significance because there is high variation within each locus. MICRO-CHECKER did not detect evidence for large allele dropout in all loci, but the software detected null alleles in some loci; however, no one locus presented null alleles in more than two populations. Thus, it is possible that *H*_*E*_ was higher than *H*_*o*_ due to presence of null alleles in some loci [84].

The *N*_*A*_ observed at each locus varied between five and 12. The loci *Tur91*, *MK6*, *MK8*, and *D22* showed the most *N*_*A*_ (10, 11, 11, and 12, respectively; Table 5), and the loci *MK9*, *D22*, *MK6*, and *MK8* showed the most mean *AR* (3.005, 3.017, 3.241, and 3.285, respectively). The loci *Tur48* and *Tur117* showed the lowest *N*_*A*_ (five and six, respectively) and the lowest mean *AR* (2.106 and 2.186, respectively). The *N*_*A*_ observed in each dolphin population ranged from two to nine, and the *AR* varied between 1.222 and 3.115 (Table 5). In general, the dolphin populations of Colombia-Honduras-PuertoRico, Cuba, and Mexico retained the most number of alleles (*N*_*A*_ ranged from two to nine) and allelic richness (*AR* varied between 1.330 and 3.115) compared with the Bocas del Toro dolphin population (*N*_*A*_ ranged from two to five, and *AR* varied between 1.226 and 2.670). In contrast, individuals from Costa Rica showed the highest allelic richness values in *TexVet5*, *Tur91*, *MK9*, and *Tur117* loci (see Table 5).

## Discussion

This study provides the first description of the population structure and genetic diversity of bottlenose dolphins from the Archipelago of Bocas del Toro (Panama) using both mtDNA-CR and microsatellite data. Our results also provide further evidence that ‘inshore’ bottlenose dolphin populations face higher risks of extinction than worldwide distributed dolphin populations. The Bocas del Toro population has a small effective population size, show high levels of genetic isolation, and are aggressively targeted on daily basis by the local dolphin-watching industry. Combined these factors can potentially reduce population viability and recovery time.

### A unique ‘inshore form’ population unit

The multiple analyses conducted here identify the Bocas del Toro dolphin population as another ‘inshore form’ in the Wider Caribbean. However, unlike other Wider Caribbean dolphin populations, it possess a unique mtDNA-CR haplotype that is grouped with other ‘inshore’ bottlenose dolphin haplotypes found in The Bahamas, Cuba, Mexico, and Puerto Rico [3, 13]. Interestingly the closest neighboring bottlenose dolphin population located 35 km north of Bocas del Toro in Gandoca-Manzanillo, Costa Rica, was identified as the WDF form. Connectivity between these two populations is likely restricted to one migrant every 10 years from Bocas del Toro to Gandoca-Manzanillo, assuming a generation time of approximately 10 years as the age at first reproduction for this species [85, 86]. These results are congruent with photo-identification data of unique natural marks on the dolphin dorsal fins taken for a span of eleven years from the Bocas del Toro dolphin population, and five years from the Gandoca-Manzanillo dolphin population. During this time, there has been no evidence of individuals moving across populations (May-Collado pers.com. 2017). Furthermore, dolphins in Bocas del Toro show higher residency [34] than those found in Gandoca-Manzanillo [87]. While several studies have found some spatial and temporal overlap between ‘inshore form’ and WDF dolphin populations (e.g., [2, 3, 9, 12, 15,17]), which does not imply genetic flow [17], such overlap is not observed in the Bocas del Toro and Gandoca-Manzanillo dolphin populations, and their establishment appear to be independent from each other.

The strong population structure of Bocas del Toro is however, not unique. Many coastal populations of bottlenose dolphins show high levels of philopatry and genetic isolation. Among these are the bottlenose dolphins from Gulf of California [15, 17], New Zealand [2], Australia [88, 89], North East Scotland [90], Ireland [91], Atlantic waters in the Iberian Peninsula [92], the Western South Atlantic Ocean [18], Gulf of Mexico [13, 14], and The Bahamas [16]. Such high philopatry may play a role in the evolution of social and ecological specializations that can lead to genetic isolation and low genetic diversity (e.g., [6, 13, 17, 89, 91, 92, 93, 94, 95, 96, 97, 98, 99]), despite no apparent geographic barriers [2, 6, 7, 9, 13, 16, 21, 88, 89, 92, 93, 94, 95, 96, 99]. Surprisingly, the Bocas del Toro dolphin population did not retain a low genetic diversity. The number of alleles and allelic richness for each microsatellite locus was not low when compared to other Caribbean populations (see Table 5). Similarly to the Caribbean, other coastal dolphin populations in the Southwestern Atlantic Ocean showed few number of alleles per locus (between one and two in *MK6*, *TexVet5*, and *Tur91* loci) [18].

Microsatellite expected heterozygosity was high (*H*_*E*_ mean = 0.53), when compared with other coastal dolphin populations such as the one found in San Antonio Bay, Argentina (*H*_*E*_ mean = 0.19) [18], but very similar to some ‘inshore’ dolphin populations in the Caribbean (such as The Bahamas) [3, 16], the Western North Atlantic [100], and the Gulf of Mexico [13, 100]. However, is important to note that the bottlenose dolphin population in the Gulf of Mexico is large (~ 12,388 dolphins just in the eastern coastal stock [101]) and it may serve as a genetic source to help maintain high genetic diversity in other ‘inshore’ populations in the Gulf of Mexico [13]. Similarly, this large population could be a genetic source for some ‘inshore’ bottlenose dolphin populations in the Caribbean, such as The Bahamas [9], which also shows higher *H*_*E*_ values [3, 9, 16]. In light of this, the moderate genetic diversity observed in Bocas del Toro may be an indication of a recent colonization event.

### Where do dolphins from Bocas de Toro come from?

Based on mtDNA-CR haplotype clustering, we identified two bottlenose dolphin population units in the Caribbean: BDT-Bahamas-Colombia-Cuba-Mexico and CostaRica-Honduras-PuertoRico. The population unit containing haplotypes from Bocas del Toro, The Bahamas, Colombia, Cuba, and Mexico contained a considerable number of individuals that were assigned to the ‘inshore form’ [3]. In contrast, the population unit formed by Costa Rica, Honduras, and Puerto Rico is formed almost exclusively by WDF individuals, since the Puerto Rico population also contains a few ‘inshore’ haplotypes. Interestingly, Puerto Rico appears to be a source of female migrants for the rest of the Caribbean [3, 102]. It is possible that the bottlenose dolphin population of Bocas del Toro is the result of a ‘founder effect’ by individuals from other coastal Caribbean dolphin populations. Evidence of such colonization have been provided by multigene analyses in the WDF in the Caribbean, the Azores and the Mediterranean Sea [3, 9], where an ancestral connection among populations worldwide seem to have been followed by founder events [2, 3, 6, 9, 10, 13, 103]. Mitogenomic data suggest that ancestral migrants of coastal bottlenose dolphins from the Western North Atlantic have colonized coastal niches in the Caribbean recently, during the late Pleistocene around 486,000 years ago [10]. It appears that the genus *Tursiops* have gone through a recent diversification in the Holocene [10], coinciding with the end of the last glacial period around 27,000 to 14,000 years ago, and low sea levels. Changes in ocean productivity and sea level, which provide new habitats by colonize [10, 104, 105], could have influenced the distribution of coastal forms [9, 10]. Geological data for the Archipelago of Bocas del Toro indicates that approximately 9,500 years ago it was part of the continent [106], and that the Archipelago as we know it today was formed around 6,000 years ago (Coates pers.com. 1997, In: [106]). Therefore, it is possible that coastal bottlenose dolphins colonized Bocas del Toro quite recently, explaining partially their current moderate genetic diversity.

### Is Bocas del Toro a dolphin population at risk?

Preliminary estimates of population size suggest that a small population of 80 bottlenose dolphins are found in Bocas del Toro (95% CI = 72–87) [34]. Our contemporary *Ne* estimated falls within this range (73 to 122) is of concern and should be considered as a measure for conservation [107], since populations with *Ne* less than 100 individuals have an increased extinction risk [83]. According to the 50/500 rule, *Ne* of 50 reproductive individuals can maintain genetic health in a population in the short term, and 500 mature individuals to the long term [83, 108]. The Bocas del Toro small dolphin population does not show evidence of an increase in size, and mortality due to accidental collisions with dolphin-watching boats may exacerbate the negative effects of genetic drift in small population size populations and potential inbreeding [83, 107].

Therefore, the combination of small dolphin population size (similar and even lower than *Ne*), genetic isolation, and intense commercial dolphin-watching activities can potentially impact the survival and fitness of the Bocas del Toro dolphin population. These dolphins sustain the largest dolphin-watching industry in Panama, interacting daily with a high number of boats [41, 109, 110], many of which do not follow national regulations [110] (Resolution ADM/ARAP NO. 01, 2007 [111]). During low tourism season, it is common to find over ten boats interacting simultaneously with the same group of dolphins [110], and a boat turnover that can 39 boats within just one hour [41]. Local operators estimate that during the high tourism season (November to March) a group of dolphins will likely interact with over 100 boats/hour [41, 110]. In 2012 at the peak of boat-dolphin interactions, seven dolphin fatalities resulted from boat collision [43]. Dolphins are also indirectly impacted by these boat encounters by significantly reducing foraging time [45]. Many of the dolphins exposed to these interactions are mothers with dependent calves [45]. Furthermore, noise levels increase with the number of boats, and although it is not clear that engine noise is masking their communicative signals, dolphins are responding to boat interactions by shifting their signal frequencies and duration [42, 112, 113], especially when interactions occurred during foraging [44]. These persistent interactions are expected to have long-term consequences in the survival and fitness of the population. In Doubtful Sound (New Zealand) dolphin-watching boats affected the dolphin’s foraging, increased their stress levels and affecting their social structure and communication [114, 115, 116, 117].

Despite the intense interactions with boats, there is no evidence that the dolphins of Bocas del Toro are moving out of this area. The cost of leaving appears to be greater than staying, even when boat traffic continues to grow. The Archipelago of Bocas del Toro provides a protected area from potential predators and rough weather, and mangroves, sea grasses, and coral reefs provide a rich source of food supply. In addition, males in this population do not appear to disperse to other areas, as is commonly described for other bottlenose dolphin populations [3, 20, 118]. The geomorphological and ecological conditions of Bocas del Toro, may be important for both males and females. Because of the pressure of the dolphin-watching industry on this population and the impending threats to the dolphins, the International Whaling Commission made four recommendations to the government of Panama to develop strategies to protect this population [119, 120, 121, 122]. Despite these recommendations, dolphin-watching industry continues to grow and impacting the dolphins at Bocas del Toro.

Similar to the situation in Bocas del Toro, there are at least 50 other dolphin populations that are small, isolated, and vulnerable to human activities [19]. Even some small dolphin populations show some philopatry in oceanic islands (e.g., [123, 124, 125, 126]), which also has conservation implications [125]. The concerns about the global IUCN categorization for bottlenose dolphins are based on the increasing evidence [26, 36, 37, 38, 114, 115, 116, 117, 127, 128] that failure to recognize local population ‘uniqueness’, and their declines can threaten the regional status and ultimately the global status of ‘common’ marine mammals, such as the bottlenose dolphins [19]. The disappearance of these local populations could take hundreds of years to be replaced by others. For the Bocas del Toro dolphin population a precautionary approach, at least until more data becomes available includes changing its national status from ‘vulnerable’ to ‘endangered’. This change could assist policy makers and resource managers to protect this population and its habitat.

## Supporting information

S1 Data Microsatellite data. (XLSX)

S1 Fig Graphic representation of Evanno et al. [71] ad hoc statistic *∆K*, which shows a clear peak in *K* = 2. (DOCX)

S1 Table Mean and Stdev *LnPK*, and *DeltaK* results for all *K* (1 to 10), according to STRUCTURE analyses. (DOCX)
