# Supplementary material for: High genetic structure and low mitochondrial diversity in bottlenose dolphins of the Archipelago of Bocas del Toro, Panama: A population at risk?
Source: PLoS One. 2017 Dec 13;12(12):e0189370. doi: 10.1371/journal.pone.0189370 (PMC5728558; doi:10.1371/journal.pone.0189370)
Supplement: S1 Fig — (DOCX) [file pone.0189370.s002.docx]

S1 Fig. Graphic representation of Evanno et al. 2005 [71] ad hoc statistic *∆K*, which show a clear peak in *K*=2.
