# Supplementary material for: High genetic structure and low mitochondrial diversity in bottlenose dolphins of the Archipelago of Bocas del Toro, Panama: A population at risk?
Source: PLoS One. 2017 Dec 13;12(12):e0189370. doi: 10.1371/journal.pone.0189370 (PMC5728558; doi:10.1371/journal.pone.0189370)
Supplement: S1 Table — (DOCX) [file pone.0189370.s003.docx]

S1 Table. Mean and Stdev *LnPK*, and *DeltaK* results for all K (1 to 10), according to STRUCTURE analyses.

| **# K** | **Mean LnP(K)** | **Stdev LnP(K)** | **Delta K** |
| --- | --- | --- | --- |
| 1 | -4235.1200 | 1.1023 | NA |
| 2 | -3454.8900 | 2.4727 | 267,317 |
| 3 | -3335.6600 | 4.8658 | 4,213 |
| 4 | -3236.9300 | 6.5630 | 5,083 |
| 5 | -3171.5600 | 7.9104 | 4,463 |
| 6 | -3141.5000 | 15.9180 | 0,064 |
| 7 | -3112.4600 | 7.9744 | 3,777 |
| 8 | -3113.5400 | 12.1209 | 1,142 |
| 9 | -3148.4700 | 32.2200 | 0,569 |
| 10 | -3145.0500 | 39.9780 | NA |
